# Embodied robots in rehabilitation-oriented interventions for older adults with disability or cognitive impairment: a scoping review

**DOI:** 10.3389/fresc.2026.1883386

**Published:** 2026-08-11

**Authors:** Wen Xu, Xian Zhou, Yu Zeng, Sixuan Han, Yanyan Hong

**Affiliations:** 1 Department of General Surgery, Nanjing Hospital of Traditional Chinese Medicine, Nanjing, China; 2 Nanjing University of Chinese Medicine, Nanjing, China; 3 Department of Nursing, Nanjing Hospital of Traditional Chinese Medicine, Nanjing, China

**Keywords:** cognitive impairment, cognitive rehabilitation, disability, exoskeletons, older adults, physical rehabilitation, rehabilitation robots, scoping review

## Abstract

**Background:**

Robots are increasingly used in later-life care. However, existing reviews often combine applications such as companionship, telepresence, medication support, monitoring, psychosocial support, traditional AI, and rehabilitation within a single body of evidence. This conceptual overlap makes it difficult to interpret the rehabilitation-specific evidence base.

**Objective:**

This study aimed to map empirical studies on embodied robots used in rehabilitation-oriented interventions for older adults with disability or cognitive impairment and to clarify the operational boundary between rehabilitation and non-rehabilitation robot applications.

**Methods:**

This scoping review followed Arksey and O'Malley’s framework, updated Joanna Briggs Institute (JBI) guidance, and PRISMA-ScR reporting principles. Six databases (i.e., PubMed, CINAHL, Web of Science Core Collection, Embase, China National Knowledge Infrastructure, and Wanfang Data) were searched from their inception to May 2026. Original empirical studies in English or Chinese were eligible if they involved embodied robots used for structured physical rehabilitation, cognitive rehabilitation, rehabilitation-oriented cognitive stimulation, or direct functional assistance with rehabilitation-relevant outcomes in older adults with disability or cognitive impairment. Consistent with a scoping review design, no formal risk-of-bias or certainty-of-evidence appraisal was conducted.

**Results:**

Eleven studies published between 2019 and 2026 were included. Six of the studies focused on cognitive rehabilitation or rehabilitation-oriented cognitive stimulation and five on physical rehabilitation. Robots served as structured cognitive facilitators, rehabilitation platforms or devices, or wearable/exoskeleton assistive systems. Study designs, comparators, intervention formats, settings, robot roles, and outcome instruments were heterogeneous. Most studies were small, preliminary, or task-specific, with generally limited follow-up. Reporting of intervention parameters and participant flow was inconsistent, and device-specific safety information was rarely distinguishable from general clinical complications.

**Conclusions:**

Applying a Rehabilitation Treatment Specification System-informed rehabilitation boundary refined the evidence map and improved conceptual interpretability. However, the included evidence is insufficient to support comparative effectiveness claims. Therefore, future studies should specify rehabilitation targets, active ingredients, mechanisms of action, intervention parameters, participant flow, follow-up, and device-specific safety profiles and should distinguish rehabilitation value from psychosocial support, companionship, acceptability, or general service delivery.

**Systematic Review Registration:**

https://osf.io/q5mrn/overview, q5mm, Open Science Framework.

## Introduction

Population ageing is increasing the number of older adults living with mobility limitations, stroke-related disability, impaired hand function, lower-limb dysfunction, mild cognitive impairment, and dementia. These conditions can reduce independence and increase rehabilitation needs (1, 2). In this review, older adults were primarily defined as persons aged 60 years or older, consistent with conventions in ageing research (1). Disability was interpreted from an International Classification of Functioning, Disability and Health perspective as diminished functioning across physical, cognitive, and participation domains, rather than as a single diagnostic label (2).

This review focuses on embodied robots, rather than traditional or disembodied AI because the primary interest is not only algorithmic prediction but also robot-mediated rehabilitation action. Traditional AI in later-life care may classify risk, generate recommendations, personalize content, or support monitoring through software systems, apps, or decision aids. In contrast, an embodied robot is a physically instantiated system that can perceive the user and task environment, reason or select context-sensitive actions, and act through speech, gesture, movement guidance, tactile feedback, force assistance, body-weight support, or other physical interactions. This perception–reasoning–acting loop is clinically important because rehabilitation often depends on repeated task practice, feedback, environmental affordances, patient effort, and therapist-supervised progression, rather than information processing alone (3, 14).

The theoretical framework used in this review was informed by the Rehabilitation Treatment Specification System (RTSS). The RTSS describes rehabilitation treatment at the component level, linking a treatment target, one or more active ingredients, and a mechanism of action, while also distinguishing proximal treatment targets from broader downstream aims (14). Using this perspective, a robot-assisted activity was considered rehabilitation-oriented only if the robot contributed to an active treatment ingredient directed at a cognition- or function-centred target through a plausible mechanism of action, and if at least one outcome reflected the target or a clinically meaningful rehabilitation aim. This framework helped distinguish robot-mediated rehabilitation from robot use for companionship, telepresence, medication management, entertainment, environmental monitoring, or general psychosocial support. Examples of valuable but non-rehabilitation robot applications include medication-management robots, assistive-robot development work, companion robots, telepresence systems, and home-support robots for chronic disease management (29–34).

Therefore, for this review, a rehabilitation-oriented robotic intervention was defined as a structured assessment, training, maintenance, or direct functional assistance programme in which an embodied robot had an explicit role in preserving or improving cognitive or physical function. This definition aimed to distinguish rehabilitation-relevant use from applications focused solely on companionship, telepresence, environmental monitoring, medication support, or general psychosocial support. While these applications may be valuable in older-adult care, they do not necessarily share the same treatment targets, active ingredients, mechanisms of action, staffing requirements, or outcome priorities as rehabilitation interventions.

The broader literature on robots and AI-supported care technologies for older adults has expanded rapidly. Existing reviews have explored AI applications for older adults with disabilities, community-care robots, and AI-enabled robots in long-term care (3–5); robot interventions for cognitive or psychological outcomes (6); telepresence robots (7, 8); PARO and social robots for behavioural, psychological, or loneliness-related outcomes (9–11); and robopets or animal-assisted/pet-robot interventions (12, 13). While these syntheses are useful for describing the wider care-robot landscape, they often combine applications with substantially different purposes, including communication support, agitation reduction, loneliness reduction, medication management, monitoring, entertainment, and rehabilitation. Such pooling can create clinical interpretability problems because psychosocial or behavioural outcomes are not equivalent to rehabilitation endpoints.

A focused scoping review is warranted to clarify what remains when the broader care-robot literature is focused on embodied robots used in rehabilitation-oriented interventions. The present review maps the available empirical studies, classifies robot roles and rehabilitation targets, and makes explicit the boundary decisions needed to distinguish rehabilitation-oriented cognitive or physical interventions from non-rehabilitation robot applications.

## Methods

### Study design

This review followed Arksey and O'Malley's scoping review framework (15). The Joanna Briggs Institute (JBI) provided updated methodological guidance for the conduct of the review (16), and reporting followed PRISMA-ScR principles (17). The review question and eligibility criteria were structured using the Population-Concept-Context framework. A scoping review design was considered appropriate because the aim was to map the rehabilitation-oriented evidence base and clarify conceptual boundaries rather than to estimate pooled effects.

A predefined protocol guided study selection, eligibility decisions, data charting, and synthesis, and the review was registered in the Open Science Framework (OSF). Consistent with the purpose of a scoping review, no formal risk-of-bias or certainty-of-evidence appraisal was undertaken. Findings are therefore presented as an evidence map and should not be interpreted as a comparative effectiveness assessment.

### Search strategy

A focused search was performed across six databases, namely, PubMed, CINAHL, Web of Science Core Collection, Embase, China National Knowledge Infrastructure (CNKI), and Wanfang Data. Searches covered each database’s inception to May 2026. Backward reference checking and limited manual searching were also used to identify additional potentially relevant studies. No separate searches of engineering databases, trial registries, or grey literature were conducted. This choice should be considered when interpreting the coverage of engineering prototypes, registry-only protocols, conference-only reports, and early feasibility studies published primarily in robotics venues.

The search strategy combined controlled vocabulary and free-text terms across four concept blocks: older adults, disability or cognitive impairment, rehabilitation robots, and rehabilitation-oriented intervention. To reduce the risk of missing boundary studies, search terms included high-level terms, such as rehabilitation robot, social robot, humanoid robot, and exoskeleton, and task-specific terms, such as hand rehabilitation robot, upper-limb rehabilitation robot, robot-assisted gait training, balance rehabilitation robot, cognitive training, and cognitive stimulation. Database-specific search strategies are provided in Supplementary Appendix 1.

### Eligibility criteria

Table 1 defines the Population-Concept-Context elements and the review-specific definition of rehabilitation-relevant outcomes.

**Table 1:** Operational eligibility criteria based on Population-Concept-Context (PCC) elements.

PCC element	Included	Excluded or not sufficient for inclusion
Population	Adults aged 60 years or older, or samples explicitly framed as older adults, with disability, functional impairment, mild cognitive impairment, dementia, stroke-related disability, gait/balance impairment, hand or upper-limb dysfunction, lower-limb dysfunction, or other rehabilitation-relevant limitations	Younger samples not framed as older adults; samples without disability, cognitive impairment, or function-centred rehabilitation need
Concept	Embodied robots with a physical robotic structure used in structured rehabilitation-oriented interventions, including physical rehabilitation, cognitive rehabilitation, rehabilitation-oriented cognitive stimulation, or direct functional assistance	Software-only AI, virtual agents, apps, sensors without an embodied robot, telepresence-only robots, medication management-only robots, entertainment-only robots, environmental monitoring, or social companionship without rehabilitation target
Context	Community, home-based, day-care, long-term care, outpatient, inpatient, rehabilitation, or other clinically relevant care settings for older adults	Non-care settings, engineering bench testing, or laboratory-only prototype testing without participant-level rehabilitation outcomes
Type of evidence	Original empirical intervention studies reporting participant-level outcomes, including randomized trials, quasi-experimental studies, pilot/feasibility intervention studies, and clinical comparative studies	Reviews, protocols, editorials, commentaries, dissertations, conference abstracts without sufficient empirical data, and full texts unavailable in English or Chinese
Rehabilitation-relevant outcomes	At least one cognition- or function-centred outcome, such as cognitive function, motor function, activities of daily living (ADL), balance, gait, upper-limb or hand function, lower-limb function, functional independence, or other rehabilitation endpoints	Satisfaction, usability, acceptability, social presence, loneliness, agitation, mood, engagement, or engineering performance alone without a cognition- or function-centred endpoint

For socially assistive robots, inclusion was deliberately conservative. A PARO, Pepper, board-game, or other socio-cognitive robotic protocol was included only when the robot delivered repeated structured tasks or therapist-/nurse-supported sessions with an explicit cognition- or function-oriented purpose and at least one rehabilitation-relevant outcome. Companion-only, affect-oriented, social presence, loneliness, agitation, satisfaction, acceptability, or engagement-only studies were excluded unless they also met the rehabilitation target and outcome criteria above. This rule was intended to reduce circularity: A structured activity and a cognition-adjacent measure alone were not sufficient unless the activity could be linked to an RTSS-compatible treatment target, active ingredient, and plausible mechanism of action.

### Study screening and boundary adjudication

All records retrieved from database searches were imported into NoteExpress for reference management. Duplicate records were removed using the software’s automated function and manual checking when necessary. Two reviewers (XZ and YZ) independently screened titles and abstracts and then independently assessed the full texts of potentially eligible articles. Reasons for exclusion at the full-text stage were recorded by category to improve transparency at the rehabilitation/non-rehabilitation boundary. Disagreements were resolved through discussion or adjudication by a third reviewer (SXH).

For routine inclusion/exclusion screening, two reviewers independently screened titles, abstracts, and full texts. Disagreements were resolved through discussion or, when needed, third reviewer adjudication. We did not calculate a *post-hoc* agreement coefficient because the protocol did not prespecify kappa, and the aim of this scoping review was evidence mapping rather than diagnostic screening accuracy. For complex borderline cases involving socially assistive robots, such as PARO, Pepper, and robot-assisted board-game protocols, standard binary screening was not the primary decision tool. These articles were flagged for focused reviewer team adjudication and assessed against the full *a priori* rehabilitation boundary rule. The reviewer team reached consensus for all flagged borderline cases after applying this rule; no unresolved disagreements remained. Accordingly, we prioritized qualitative transparency over numerical reliability metrics for this specific subset. Table 2 reports the *a priori* rule, classification, and final consensus rationale.

**Table 2:** *A priori* boundary rule and adjudication of borderline robot applications.

Study/robot type	Structured protocol?	RTSS-compatible target	Rehabilitation-relevant outcome	Classification	Adjudication rationale
Chen et al., PARO (22)	Yes	Cognitive/functional stimulation in group sessions	MMSE and finger tapping, plus autonomic and wellbeing measures	Included	Retained because the PARO protocol was embedded in repeated, structured sessions and reported cognition/function-adjacent outcomes; not interpreted as evidence for companionship-only use
Figliano et al., Pepper (23)	Yes	Structured cognitive and socio-cognitive facilitation	Autonomy, focus, ACE-R/MMSE profile, feasibility/engagement	Included as preliminary evidence	Retained because Pepper delivered structured tasks with cognitive/socio-cognitive aims; interpreted cautiously because effectiveness outcomes were limited
Lin et al., robot-assisted board games (21)	Yes	Cognitive training/stimulation through repeated interactive tasks	MMSE, ADAS-Cog, NPI, GDS, UCLA loneliness, usability	Included	Retained because the intervention involved repeated cognition-focused tasks and measured cognition; mental health outcomes were treated as secondary
Companion-only social robot studies	No or unclear	No explicit rehabilitation target	Loneliness, social presence, satisfaction, or acceptability only	Excluded	Excluded when the robot primarily provided companionship or social engagement without a cognition- or function-centred target and rehabilitation-relevant outcome
Telepresence-only robot studies	No rehabilitation treatment component	Communication/social connection rather than treatment target	Communication, caregiver connection, feasibility	Excluded	Excluded because telepresence supports service delivery or communication but does not itself deliver a rehabilitation treatment component

The first three rows represent specific included borderline studies, whereas the final two rows illustrate excluded intervention classes, classified according to the same *a priori* rule.

### Data extraction and synthesis

Data charting was conducted independently using a structured extraction form. Charted items included publication year, country or region when available, target population and setting, robot type and role, rehabilitation target, study design, comparator, intervention dose when available, follow-up, reported outcomes, adherence or withdrawal information when available, adverse events or safety reporting when available, funding information when available, and the main interpretation relevant to evidence mapping. Some fields remained variably documented across studies because intervention dose, participant descriptors, funding information, and safety outcomes were not consistently reported in the primary literature.

Given the heterogeneity of the evidence, findings were synthesized narratively rather than statistically. Studies were organized first by rehabilitation target (cognitive rehabilitation or rehabilitation-oriented cognitive stimulation vs. physical rehabilitation) and then interpreted according to robot role (structured cognitive facilitator, rehabilitation platform or device, or wearable/exoskeleton assistive system). Reporting patterns for intervention dose, follow-up, adherence, withdrawals, and safety were summarized descriptively. No formal risk-of-bias or certainty appraisal was undertaken; therefore, the results should be interpreted as an evidence map rather than a comparative assessment of effectiveness.

## Results

### Study selection process and results

**Figure 1:**
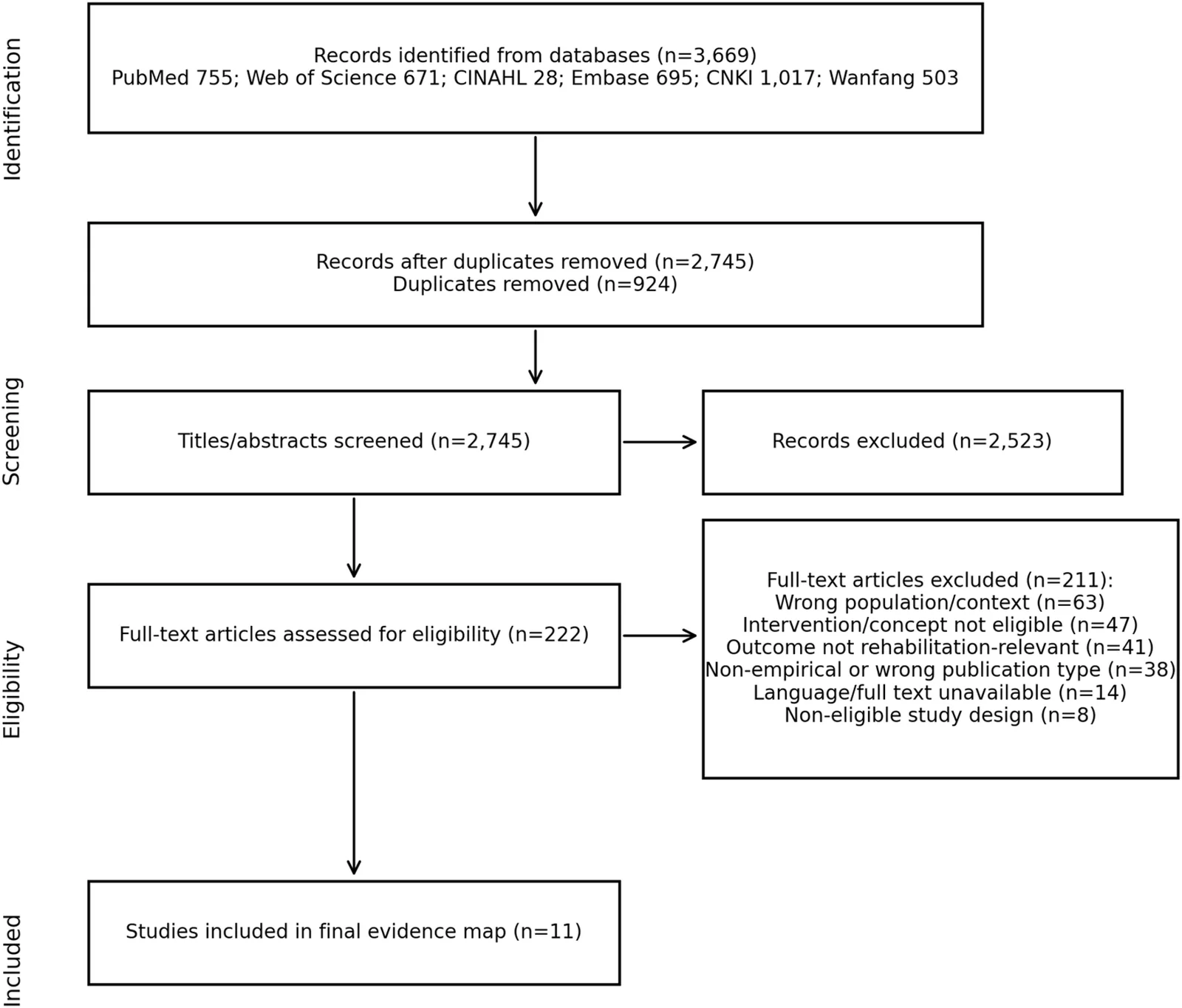
PRISMA-ScR-style study selection flow diagram.

Database searches identified 3,669 records. After removing 924 duplicates, 2,745 titles and abstracts were screened, which led to the exclusion of 2,523. A total of 222 full-text articles were assessed for eligibility, and 211 were excluded for not meeting the criteria regarding population, concept, intervention, outcome, publication type, language, or full-text availability. Finally, 11 studies published between 2019 and 2026 were included in the evidence map. Figure 1 presents the PRISMA-ScR-style flow diagram. A record-level list of full-text exclusions with reasons is provided in Supplementary Appendix 3. The core characteristics and reporting features of the included studies are summarized in Table 3.

**Table 3:** Core characteristics and reporting features of included studies.

Study	Country/setting	Population	Robot/target	Design and dose/follow-up	Outcome measures/tools
Park et al. (18)	South Korea; community	Older adults with mild cognitive impairment; *n* = 143	Sil-Bot; cognitive training	Randomized controlled trial; 6 weeks; follow-up unclear	Cognitive and depression-related outcomes
Lee et al. (19)	South Korea; memory clinic/home	Adults ≥60 years with mild cognitive impairment; *n* = 46 randomized; *n* = 41 analysed	Bomy; home cognitive training	Rater-blind randomized controlled trial; 4 weeks; no long-term follow-up	CANTAB and other cognitive/depression measures
Lim and Oh (20)	South Korea; day-care centres	Mild-to-moderate dementia; *n* = 66	PIO; group cognitive stimulation	Randomized controlled trial; 6 weeks; immediate post-test	Cognitive function and depression
Lin et al. (21)	Taiwan; long-term care/day-care centres	Older adults with mild cognitive impairment; *n* = 109	Board-game robot platform; cognitive tasks	Cluster/quasi-experimental design; 12 weeks; 3-month follow-up	MMSE; ADAS-Cog; GDS-15; NGSES; SWLS; SUS
Chen et al. (22)	Taiwan; dementia day-care centres	Mild dementia, age ≥65 years; *n* = 118	PARO; group cognitive/functional stimulation	Two-arm randomized controlled trial; 6 weeks; 1-month follow-up	MMSE; FTT; HRV; GDS-SF; UCLA-LS; WEMWBS
Figliano et al. (23)	Italy; day-care centre	Mild-to-moderate dementia; *n* = 9	Pepper; cognitive/socio-cognitive facilitation	Feasibility/acceptability study; 4 weeks; no comparator	Acceptability, engagement, focus, autonomy; ACE-R/MMSE profile
Radder et al. (24)	Netherlands/Sweden/Switzerland; home	Hand function decline; *n* = 91	ironHand glove; hand assistance/training	Pilot randomized clinical study; 4 weeks	Maximal pinch grip; BBT; JTHFT; handgrip strength; SUS
Zhang and Xu (25)	China; hospital	Elderly stroke patients; *n* = 80	BURT upper-limb robot; upper-limb task/game training	Randomized clinical intervention; 4 weeks	FMA-UE; FMA-SE; FMA-WH; BI
Liu et al. (26)	China; hospital	Lower-limb dysfunction after burns; *n* = 90	XYKXZK-9 lower-limb feedback robot; gait training nursing	Randomized clinical intervention; 8 weeks	Gait parameters; 6MWT; FAC; BBS; FMA-LE
Jin et al. (27)	China; hospital	ICU-acquired weakness; *n* = 114	Lower-limb exoskeleton; adjunct physical rehabilitation	Prospective randomized trial; dose not fully reported	CPAx; MRC muscle strength scale; BI; muscle-quality imaging; BMD; serum markers; complications
Cui et al. (28)	China; hospital	Lower-limb fracture plus type 2 diabetes; *n* = 150	Balance-disorder robot; balance/gait training with 3D feedback	Clinical comparative intervention; 12 weeks	BBS; ABC; FAC; FIM; gait measures; FMA-LE; LEFS; GQOLI-74

Outcome measure/tool abbreviations: ABC, Activities-specific Balance Confidence Scale; ACE-R, Addenbrooke's Cognitive Examination-Revised; ADAS-Cog, Alzheimer's Disease Assessment Scale-Cognitive Subscale; BBT, Box and Block Test; BI, Barthel Index; BBS, Berg Balance Scale; BMD, bone mineral density; CANTAB, Cambridge Neuropsychological Test Automated Battery; CPAx, Chelsea Critical Care Physical Assessment Tool; FAC, Functional Ambulation Category; FIM, Functional Independence Measure; FMA, Fugl–Meyer Assessment; FMA-LE, Fugl–Meyer Assessment Lower-Extremity Component; FMA-SE, Fugl–Meyer Assessment Shoulder–Elbow Component; FMA-UE, Fugl–Meyer Assessment Upper-Extremity Component; FMA-WH, Fugl–Meyer Assessment Wrist–Hand Component; FTT, finger tapping test; GDS-15, Geriatric Depression Scale-15; GDS-SF, Geriatric Depression Scale-Short Form; GQOLI-74, Generic Quality of Life Inventory-74; HRV, heart rate variability; JTHFT, Jebsen–Taylor Hand Function Test; LEFS, Lower Extremity Functional Scale; MMSE, Mini-Mental State Examination; MRC, Medical Research Council; NGSES, New General Self-Efficacy Scale; NPI, Neuropsychiatric Inventory; SUS, System Usability Scale; SWLS, Satisfaction with Life Scale; UCLA-LS, University of California Los Angeles Loneliness Scale; WEMWBS, Warwick–Edinburgh Mental Wellbeing Scale; 6MWT, 6-Min Walk Test. Device-related adverse events, adherence, withdrawals, and longer-term follow-up were not consistently reported across studies; absence of reporting should not be interpreted as absence of events.

### Characteristics of the included studies and interventions

Application of the rehabilitation-oriented eligibility criteria reduced the final corpus to a small, conceptually more focused set of studies. Six studies addressed cognitive rehabilitation or rehabilitation-oriented cognitive stimulation, and five addressed physical rehabilitation. Settings included home-based rehabilitation, community programmes, dementia day-care centres, long-term care or day-care facilities, group-based care settings, centre-based cognitive intervention settings, and rehabilitation-related clinical environments.

Across the 11 included studies, robots served three broad roles: (1) structured cognitive facilitators, usually humanoid or socially assistive systems embedded in repeated task-based intervention sessions; (2) rehabilitation platforms or devices used for specific motor or cognitive tasks; and (3) wearable or exoskeleton assistive systems used in task-specific physical rehabilitation. The evidence remained heterogeneous with respect to participant characteristics, country or region, intervention format, comparator, outcome selection, funding information, adherence reporting, withdrawal reporting, and follow-up duration. Four of the five physical rehabilitation studies were conducted in Chinese hospital settings, an important feature of the evidence map that limits the generalizability of the physical rehabilitation subgroup.

### Cognitive rehabilitation and rehabilitation-oriented cognitive stimulation

Six studies examined robot-assisted cognitive rehabilitation or rehabilitation-oriented cognitive stimulation in older adults with mild cognitive impairment or mild-to-moderate dementia. In this review, structured cognitive training refers to repeated, standardized cognitive tasks with explicit training goals. Rehabilitation-oriented cognitive stimulation refers to structured stimulation with cognition- or function-centred targets but broader social or socio-cognitive content. Cognitive rehabilitation is used as the umbrella term for intervention approaches intended to improve or preserve cognitive functioning.

Park et al. evaluated the humanoid robot Sil-Bot in a structured cognitive training programme for community-dwelling older adults with mild cognitive impairment (18). Lee et al. evaluated a 4-week home-based robot cognitive intervention in patients with mild cognitive impairment and reported selected cognition-related outcome changes (19). Lim and Oh examined a programme using PIO, a parrot-type socially assistive robot, for older adults with mild-to-moderate dementia and reported cognition- and depression-related outcomes (20). Lin et al. described robot-assisted board games for older adults with mild cognitive impairment and reported cognitive and selected mental health-related outcomes (21).

Two studies required particularly careful boundary classification. Chen et al. used a group-based PARO protocol in older adults with mild dementia and reported cognitive, autonomic, and mental wellbeing outcomes (22). Figliano et al. tested Pepper-delivered structured cognitive and socio-cognitive sessions in older adults with mild-to-moderate dementia and primarily reported signals of feasibility, engagement, autonomy, focus, and qualitative acceptability (23). These studies were retained because the robot was embedded in an explicit, structured protocol with cognition- or function-related outcomes or cognition-oriented stimulation aims. They should not be interpreted as evidence for companionship-only or affect-oriented social robot applications.

### Physical rehabilitation

Five studies addressed physical rehabilitation in functionally impaired older adults. Radder et al. evaluated home rehabilitation supported by a wearable soft-robotic device for improving hand function in older adults (24). Zhang and Xu evaluated robot-assisted upper-limb rehabilitation training for older adults with stroke (25). Liu et al. described robot-assisted gait training nursing for older adults with lower-limb dysfunction after burns (26). Jin et al. reported on lower-limb exoskeleton rehabilitation in older adults with ICU-acquired weakness (27). Cui et al. examined robot-assisted balance-disorder rehabilitation training for older adults with lower-limb fracture complicated by type 2 diabetes (28).

The three Chinese physical rehabilitation citations previously listed under “Journal of Robotic Surgery” have been corrected to *Chinese Journal of Robotic Surgery*. DOI information has been added for references (25, 26, 28).

Compared with the cognitive studies, the physical rehabilitation studies were easier to classify clinically because they targeted specific endpoints such as hand function, upper-limb motor function, gait, balance, lower-limb motor recovery, or functional independence. However, these studies remained task-specific, short-term, and heterogeneous in terms of intervention dose, comparators, and outcome instruments.

### Distribution of evidence across domains

Overall, the evidence map suggests that rehabilitation-oriented robots for older adults are concentrated in clearly delineated use cases: structured cognitive training or stimulation, hand and upper-limb rehabilitation, gait and balance rehabilitation, and exoskeleton-assisted lower-limb recovery. While this focused boundary improves conceptual coherence, it does not eliminate methodological heterogeneity.

Intervention dose reporting was incomplete or inconsistent. Some studies provided duration, frequency, and session length, while others reported only total duration or broad training procedures. At least one study lacked sufficient detail for standardized comparison. Where reported, intervention periods generally ranged from 4 to 12 weeks. However, because robot type, clinical target, session frequency, comparator, and outcome measures differed substantially, dose–response interpretation was not possible.

Safety, adverse events, adherence, and withdrawals were also insufficiently reported. In several studies, safety or device-related adverse events were either not reported in the available manuscript or not clearly separated from general clinical complications. Therefore, the absence of safety reporting should not be interpreted as evidence that no adverse events occurred. This reporting gap is particularly important because rehabilitation robots are embodied and may involve physical interaction, repetitive movement, body-weight support, exoskeleton assistance, or cognitively demanding interaction protocols.

## Discussion

This scoping review mapped a deliberately focused subset of the older-adult robot literature: embodied robots used in rehabilitation-oriented interventions for older adults with disability or cognitive impairment. The principal contribution is conceptual clarification rather than proof of effectiveness. Applying a strict rehabilitation-oriented boundary reduced the final corpus to 11 studies and showed that the literature clusters mainly around two rehabilitation targets: cognitive intervention or stimulation and task-specific physical rehabilitation.

This finding helps explain why broader reviews of care robots can be difficult to interpret clinically. For example, reviews of AI-enabled care robots and community-care robots provide important service-level context (3–5). Similarly, reviews of robot interventions for cognitive or psychological outcomes and telepresence robots describe overlapping psychosocial or care-support evidence (6–8). Reviews focusing on PARO and social robots highlight behavioural or loneliness-related applications (9–11). Furthermore, reviews of robopet and animal-assisted/pet robots further describe mixed-purpose applications in older-adult care (12, 13). However, these diverse literatures often combine various applications such as communication support, companionship, behavioural symptom management, monitoring, medication support, and rehabilitation. In contrast, rehabilitation-oriented robot use requires an explicit cognition- or function-centred target, a structured intervention protocol, and outcomes meaningful for cognitive function, motor recovery, ADL, gait, balance, or other rehabilitation endpoints.

Using the RTSS clarified this boundary. The review did not consider embodiment alone as sufficient evidence of rehabilitation relevance. Instead, the robot had to contribute to an active ingredient—such as guided practice, feedback, prompting, task progression, movement assistance, or structured cognitive stimulation—directed at a defined treatment target through a plausible mechanism of action (14). This approach also explains why an affective or socially engaging robot might be included in one context but excluded in another: The deciding factor was not the robot platform itself, but the treatment component in which it was embedded.

The cognitive domain proved more conceptually heterogeneous than the physical domain. Some studies resembled structured cognitive training (18–20), whereas robot-assisted board-game, PARO, and Pepper protocols were better interpreted as rehabilitation-oriented cognitive stimulation or socio-cognitive facilitation (21–23). Therefore, borderline studies using PARO, Pepper, or robot-assisted board games should be interpreted cautiously. Their inclusion depends on the presence of repeated structured tasks, an RTSS-compatible cognition- or function-centred target, and rehabilitation-relevant outcomes, not merely on the use of a socially assistive robot.

The physical rehabilitation studies, while clinically more straightforward, remained task-specific and heterogeneous. They addressed hand function (24), upper-limb recovery after stroke (25), gait dysfunction after burns (26), ICU-acquired weakness (27), and balance or gait recovery after lower-limb fracture complicated by type 2 diabetes (28). Four of these five physical rehabilitation studies were conducted in Chinese hospital settings. This concentration may reflect active clinical adoption of rehabilitation robots in China, but it also denotes that service models, staffing patterns, devices, and outcome reporting may not generalize to other health systems.

For clinical implementation, robot deployment should remain problem-led rather than technology-led. A humanoid or socially assistive robot may be appropriate when the goal is structured cognitive training or cognitive stimulation under supervision. A wearable soft-robotic device may be more relevant for hand function assistance or training. Upper-limb, gait, balance, and exoskeleton systems may be more relevant in therapist- or nurse-supervised physical rehabilitation pathways. In all cases, the robot should supplement a clearly defined care pathway rather than substitute for clinical reasoning, safety monitoring, or individualized rehabilitation planning.

The review also identifies reporting gaps that limit translation. Intervention dose, comparator conditions, adherence, withdrawals, follow-up, and funding information were not consistently reported. Safety reporting was particularly limited: Several studies either did not report adverse events or did not distinguish device-related events from general medical complications. For embodied robots, this distinction is essential because the intervention may involve repetitive movement, physical contact, resistance, body-weight support, exoskeleton assistance, or cognitively demanding human–robot interaction. Future studies should separately report device-related adverse events, near misses, user fatigue, pain, falls, skin problems, device stoppages, and withdrawals attributable to the robot or training protocol.

This review has several limitations. First, the search strategy focused on health, nursing, rehabilitation, and Chinese biomedical databases, but did not separately search engineering databases, trial registries, or grey literature sources. Therefore, early engineering feasibility studies, conference proceedings, registry-only protocols, and unpublished device evaluations may have been missed. Second, because this was a scoping review, no formal critical appraisal or certainty-of-evidence assessment was undertaken. Third, the final map was small and heterogeneous, preventing conclusions about comparative effectiveness, optimal robot type, dose–response relationships, or durability of effects. Fourth, some included Chinese-language studies had limited indexing in international databases; corrected journal titles and available identifiers have therefore been provided to improve verifiability.

## Conclusion

Embodied robots used in rehabilitation-oriented interventions for older adults with disability or cognitive impairment are currently concentrated in two main domains: robot-assisted cognitive intervention or stimulation and task-specific physical rehabilitation. Applying an RTSS-informed rehabilitation boundary clarifies why some social, companion, telepresence, monitoring, or medication management robot uses were excluded, despite their potential value in older-adult care. The current evidence map suggests short-term cognition- or function-related signals in selected contexts; however, heterogeneous designs, limited follow-up, and incomplete reporting of intervention parameters and safety profiles preclude conclusions about comparative effectiveness, long-term impact, or clinical scalability. Future research should align robot use with clearly specified treatment targets, active ingredients, mechanisms of action, and standardized function-centred endpoints.

## Data Availability

The original contributions presented in the study are included in the article/Supplementary Material, further inquiries can be directed to the corresponding author.
