# Supplementary material for: Embodied robots in rehabilitation-oriented interventions for older adults with disability or cognitive impairment: a scoping review
Source: Front Rehabil Sci. 2026 Aug 11;7:1883386. doi: 10.3389/fresc.2026.1883386 (PMC13503602; doi:10.3389/fresc.2026.1883386)
Supplement: Supplementary file 1 [file Supplementaryfile1.docx]

**Supplementary Appendix 1. Database-specific search strategies**

**PubMed**

(("Aged"[Mesh] OR aged OR older adult* OR older people OR elder* OR elderly OR geriatric*) AND
(dementia OR "mild cognitive impairment" OR "cognitive decline" OR disab* OR "functional impairment" OR stroke OR gait OR balance OR "hand function" OR "upper limb" OR "lower limb") AND
("rehabilitation robot*" OR exoskeleton* OR "robot-assisted gait training" OR "upper-limb rehabilitation robot*" OR "hand rehabilitation robot*" OR "balance rehabilitation robot*" OR "cognitive training robot*" OR humanoid robot* OR social robot* OR Pepper OR PARO OR PIO OR "Sil-Bot") AND
(rehabilitation OR "rehabilitation-oriented intervention" OR "cognitive rehabilitation" OR "cognitive stimulation" OR "physical rehabilitation" OR "functional recovery" OR "activities of daily living" OR ADL))

Embase, CINAHL, Web of Science Core Collection, CNKI, and Wanfang Data used equivalent controlled-vocabulary and free-text terms translated or adapted to each database. Chinese search blocks included 老年人/老年患者/高龄/老年, 轻度认知障碍/痴呆/认知下降/功能障碍/卒中/步态/平衡/手功能, 康复机器人/外骨骼机器人/机器人辅助步态训练/上肢康复机器人/手功能康复机器人/平衡康复机器人/认知训练机器人/社交机器人/人形机器人, and 康复/认知康复/认知刺激/功能恢复/步态训练/平衡训练/日常生活能力.
