# Supplementary material for: Embodied robots in rehabilitation-oriented interventions for older adults with disability or cognitive impairment: a scoping review
Source: Front Rehabil Sci. 2026 Aug 11;7:1883386. doi: 10.3389/fresc.2026.1883386 (PMC13503602; doi:10.3389/fresc.2026.1883386)
Supplement: Supplementary file 2 [file Supplementaryfile2.docx]

**Supplementary Appendix 2. Full-text exclusion category definitions and counts**

| **Category used in revised flow diagram** | **n** | **Definition** |
| --- | --- | --- |
| Wrong population/context | 63 | Population not older adults or not framed as older adults; setting or population not relevant to disability, cognitive impairment, or rehabilitation need. |
| Intervention/concept not eligible | 47 | No embodied robot; software-only AI; sensor-only monitoring; telepresence-only, medication-management-only, entertainment-only, or companion-only robot use. |
| Outcome not rehabilitation-relevant | 41 | No cognition- or function-centred rehabilitation endpoint; outcomes limited to usability, satisfaction, acceptability, social presence, loneliness, agitation, engagement, or engineering performance. |
| Non-empirical or wrong publication type | 38 | Review, protocol, editorial, commentary, conference abstract without sufficient empirical data, dissertation, or non-journal report retrieved during citation checking/manual search. |
| Language/full text unavailable | 14 | Full text unavailable or not available in English or Chinese. |
| Non-eligible study design | 8 | Article did not provide extractable participant-level intervention data or did not meet empirical intervention-design criteria; this is an eligibility label, not a quality appraisal. |
